# Metabolic Changes in *Synechocystis* PCC6803 upon Nitrogen-Starvation: Excess NADPH Sustains Polyhydroxybutyrate Accumulation

**DOI:** 10.3390/metabo3010101

**Published:** 2013-02-06

**Authors:** Waldemar Hauf, Maximilian Schlebusch, Jan Hüge, Joachim Kopka, Martin Hagemann, Karl Forchhammer

**Affiliations:** 1 Interfakultäres Institut für Mikrobiologie und Infektionsmedizin Tübingen, Eberhard-Karls-Universität Tübingen, Auf der Morgenstelle 28, Tübingen, 72070, Germany; E-Mails: waldemar.hauf@gmail.com (W.H.); maximilian.schlebusch@googlemail.com (M.S.);; 2 Max-Planck-Institut für Molekulare Pflanzenphysiologie, Am Mühlenberg 1, Golm, 14476, Germany; E-Mails: huege@ipk-gatersleben.de (J.H.); kopka@mpimp-golm.mpg.de (J.K.); 3 Universität Rostock, Institut Biowissenschaften, Pflanzenphysiologie, Albert-Einstein-Str. 3, Rostock, D-18059, Germany; E-Mail: martin.hagemann@uni-rostock.de

**Keywords:** cyanobacteria, metabolome, nitrogen starvation, sorbitol, NADPH/NADP ratio, redox balance, Polyhydroxybutyrate, PHB synthase

## Abstract

Polyhydroxybutyrate (PHB) is a common carbon storage polymer among heterotrophic bacteria. It is also accumulated in some photoautotrophic cyanobacteria; however, the knowledge of how PHB accumulation is regulated in this group is limited. PHB synthesis in *Synechocystis* sp*.* PCC 6803 is initiated once macronutrients like phosphorus or nitrogen are limiting. We have previously reported a mutation in the gene *sll0783* that impairs PHB accumulation in this cyanobacterium upon nitrogen starvation*.* In this study we present data which explain the observed phenotype. We investigated differences in intracellular localization of PHB synthase, metabolism, and the NADPH pool between wild type and mutant. Localization of PHB synthase was not impaired in the *sll0783* mutant; however, metabolome analysis revealed a difference in sorbitol levels, indicating a more oxidizing intracellular environment than in the wild type. We confirmed this by directly measuring the NADPH/NADP ratio and by altering the intracellular redox state of wild type and *sll0783* mutant. We were able to physiologically complement the mutant phenotype of diminished PHB synthase activity by making the intracellular environment more reducing. Our data illustrate that the NADPH pool is an important factor for regulation of PHB biosynthesis and metabolism, which is also of interest for potential biotechnological applications.

## 1. Introduction

Cyanobacteria are Gram-negative prokaryotes capable to perform oxygenic photosynthesis [1]. They thrive in almost all illuminated ecosystems and contribute to the global carbon-cycle of the biosphere. In many natural environments, the availability of phosphorus and combined nitrogen is growth-limiting and, therefore, microorganisms have evolved various mechanisms to cope with this constraint [2]. Unicellular non-diazotrophic cyanobacteria respond to nitrogen limitation by a process termed chlorosis [3]. In this process, the light harvesting complexes are degraded and photosynthetic activity declines concomitant with degradation of thylakoid membranes [4]. The response towards changing levels of combined nitrogen in the environment and physiological adaptation to these conditions is mediated by the NtcA- and PII-system which induces alteration of gene expression as well as metabolic adaptation to altered nutrient availability [5,6,7].

During nitrogen starvation, carbon polymers like glycogen [4] and in some species PHB are accumulated [8]. The genes coding for precursor biosynthesis of PHB in *Synechocystis* sp. PCC 6803 are known; β-ketothiolase PhaA (*slr1993*), which condenses two acetyl-CoA to acetoacetyl-CoA, and acetoacetyl-CoA reductase PhaB (*slr1994*), which is responsible for reducing acetoacetyl-CoA with NADPH to hydroxybutyryl-CoA, are organized in one operon [9]. PHB synthase, the enzyme catalyzing the polymerization reaction to polyhydroxybutyrate, is encoded in a second operon and forms a heterodimer of PhaE (*slr1829*) and PhaC (*slr1830*) [10]. Expression of both operons is up-regulated upon nitrogen starvation [11] but biosynthetic activation of PHB synthase is independent of protein biosynthesis [12]. Hence, a complex regulatory network, which integrates different input signals, controls PHB biosynthetic activity and PHB granule formation.

How PHB granules form is still a matter of debate and several hypothesis have been proposed to explain experimental observations [13,14]. One of them is the budding model, which claims that PHB synthase associates with the cytoplasmic membrane and synthesizes PHB in the hydrophobic part of the membrane. This model requires PHB synthase to be spatially regulated within a cell. Thus, a mechanism that controls intracellular localization has to be present.

We have previously reported that inactivation of *sll0783*, a highly induced gene upon nitrogen starvation [15], results in a phenotype with highly reduced PHB accumulation. After induction of nitrogen starvation, PHB synthase was initially activated but decayed rapidly during prolonged nitrogen starvation [11]. Since the expression of the *pha*-genes was not strongly affected in the mutant, and the level of acetyl-CoA was not decreased, we suspected that impaired PHB synthase activity is due to a posttranscriptional process, in which the product of *sll0783* is involved. In this work we further investigated the cause of ceasing PHB synthase activity in the *sll0783* mutant by assessing the intracellular localization of PHB synthase and globally analyzing changes in metabolism upon nitrogen starvation.

## 2. Results and Discussion

As shown previously, impaired PHB accumulation in an insertion knock out of *sll0783* in *Synechocystis* sp. PCC 6803 is due to a decay of transiently induced PHB synthase activity [11]. In the enzymatic assays of PHB synthase activity, we observed that the activity of the enzyme was mostly present in the insoluble fraction and only a minor activity could be detected in the soluble fraction after onset of nitrogen starvation. With prolonged nitrogen starvation, the biosynthetic activity of PHB synthase in the insoluble fraction of the *sll0783* mutant had almost vanished. Since the budding model for PHB granule biogenesis requires the PHB synthase to change its intracellular localization from a soluble to a membrane localized state, we asked the question whether the impaired activity of PHB synthase in the *sll0783* mutant is related to impaired localization of the enzyme as compared to the wild type.

### 2.1. Intracellular Localization of PHB Synthase

To resolve PHB localization in wild type and *sll0783* mutant cells, recombinant reporter strains were constructed with translational fusions of both PHB synthase subunits to eGfp to study accumulation and localization of PHB synthase upon nitrogen starvation. The genes *phaE* (*slr1829*) and *phaC* (*slr1830*) together with each 100 bp promoter upstream region were amplified by PCR with genomic DNA from *Synechocystis*. The gene encoding eGfp was amplified from plasmid pCESL19 [16] by PCR. Translational fusions of PhaE- and PhaC-eGfp were generated by performing a long flanking homology PCR and cloning the resulting PCR products in the broad host range vector pVZ322 [17]. The construct integrity was verified by sequencing and the vectors pVZ322-1829 (encoding PhaE-eGfp) and pVZ322-1830 (encoding PhaC-eGfp) were transformed in the wildtype and *sll0783* mutant background by triparental mating [18].

Additionally, we determined whether the translational fusion of eGfp at the C-terminus of either subunit of PHB synthase impaired localization and biosynthetic activity. We transformed both constructs in a mutant background in which either PhaE or both PhaE and PhaC were deleted by insertion of a kanamycin resistance cassette in their coding sequences. In both cases, the eGfp-tagged versions of PhaE and PhaC were able to complement the knock out, demonstrating that the fusion proteins were functional. PHB accumulation was observed once cells were starved for nitrogen and visualized using nile red. The eGfp signal co-localized with the nile red signal (as indicated by the orange color in the overlay) demonstrating that the tag did not alter the intracellular localization of PHB synthase to PHB granules (supplementary Figure S2). PHB granule numbers per cell and granule diameters were not affected.

**Figure 1:**
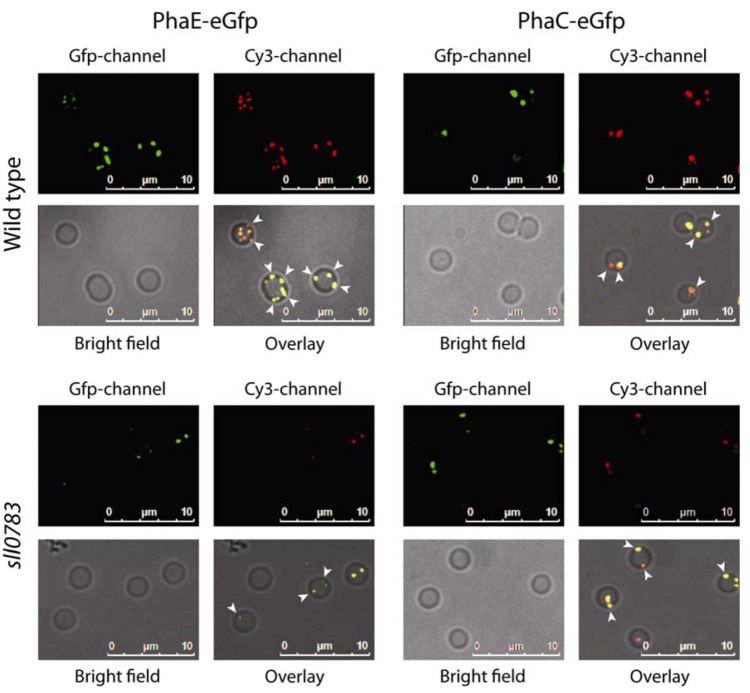
Intracellular localization of PhaC-eGfp and PhaE-eGfp in wild type and the *sll0783* mutant. Polyhydroxybutyrate (PHB) granules were stained with nile red and co-localization of both proteins to PHB granules was monitored. Co-localization between eGfp and nile red is seen as orange in the overly of all three channels. White arrowheads point to PHB granules which are localized to the cell periphery close to the cytoplasmic membrane as visualized by Bright field microscopy.

Since the C-terminal fusion did not alter PHB biosynthetic activity and intracellular localization, positioning of PHB synthase was monitored in wild type and *sll0783* mutant background after cells had been transferred to BG11-medium lacking a combined nitrogen source. Cells were sampled 24 h, 48 h and 120 h after nitrogen starvation was induced. PHB granules were mostly observed in the periphery (white arrowheads) of the cell and granule number increased throughout nitrogen starvation in the wild type. As seen previously, the *sll0783* mutant accumulated less PHB [11] and PHB granules were small even after 120 h of nitrogen starvation as seen in Figure 1. Nevertheless, both PHB synthase subunits co-localized with the residual PHB granules ruling out the possibility that impaired localization of PHB synthase could be the cause of deficient PHB synthase activity in the *sll0783* mutant. These results were confirmed by western blot analysis showing similar accumulation and distribution of PhaE between soluble and insoluble fraction in wild-type and *sll0783* mutant prior and during nitrogen starvation (supplementary Figure S3). (Please change Figure numbers into right order)

### 2.2. Metabolome Analysis of Wild Type Synechocystis sp. PCC 6803 and sll0783 Mutant

Since intracellular localization of PHB synthase was not altered in the *sll0783* mutant, we focused on the metabolic aspect of PHB biosynthesis to elucidate the cause for the observed phenotype. Our previous results showed that acetyl-CoA levels were elevated in the mutant compared to the wild type. Hence, we investigated the metabolic profiles of 39 metabolites by a non-targeted GC-MS based metabolic profiling of wild type *Synechocystis* and *sll0783* mutant upon nitrogen starvation. Time points were chosen to monitor rapid acclimation towards nitrogen starvation within 24 h and long term adaptation was covered by the last time point, when cells have experienced 1 week of nitrogen starvation. Prior to sampling for metabolite extraction, cells were transferred in medium lacking a nitrogen source and were harvested by rapid filtration at different time points during nitrogen starvation. Harvested cells were immediately frozen in liquid nitrogen to stop all biochemical reactions and metabolites were extracted as previously described [19]. All data presented is the average of three independent biological samples. The metabolic profiling covered the central C and N metabolism, which is dominated by the Calvin-Benson cycle and the associated 2-phosphoglycolate metabolism as well as glycolysis, OPP, and the TCA cycle [20]. The TCA cycle is the interface between carbon and nitrogen metabolism since its intermediate 2-oxoglutarate provides carbon backbones for amino acid biosynthesis via the GS-GOGAT cycle [21].

#### 2.2.1. Amino Acids

**Figure 2:**
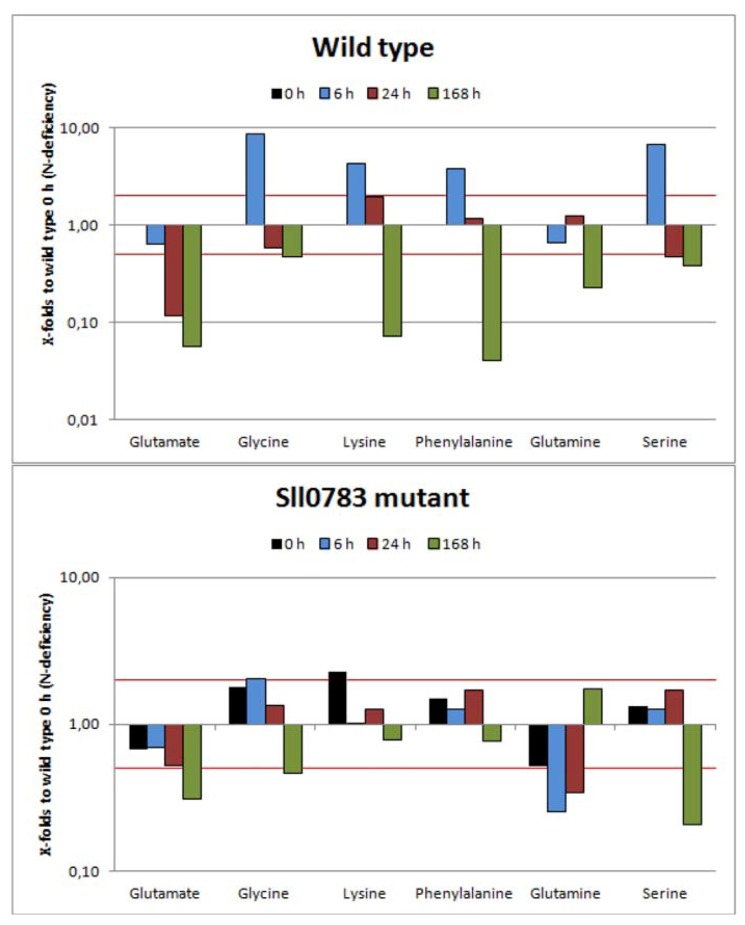
Amino acid levels of the wild type and the *sll0783* mutant throughout nitrogen starvation. Black bars represent the metabolites levels prior to nitrogen starvation (0 h), cyan bars represent values obtained 6 h, red bars 24 h and green bars 168 h after nitrogen starvation has been induced. All bars represent x-fold changes in metabolite levels compared to the level in wild type at time point 0 h. The red lines mark the values 2 and 0.5. Values above or below these lines represent statistically significant changes of metabolite levels when compared to wild type at time point 0 h.

Within the first 24 h of nitrogen starvation, *Synechocystis* undergoes a dramatic change in metabolism [3]. In the first 6 h of nitrogen starvation, intracellular amino acid concentrations increase above levels detected prior to nitrogen starvation as seen in Figure 2. Within 24 h, the intracellular concentration of each amino acid drops and is similar to values obtained prior to the onset of nitrogen starvation. With prolonged nitrogen deprivation intracellular levels of amino acids decrease dramatically, for example, up to 10 times less lysine or phenylalanine is available compared to conditions when biologically available nitrogen is abundant. Glutamine and glutamate are an exception to this behavior. The intracellular levels of both amino acids decrease steadily throughout nitrogen starvation and do not peak at 6 h as observed for other amino acids. The amino acid pools of wild type and the *sll0783* mutant differ only slightly prior to nitrogen starvation and show similar tendencies after cells were deprived of nitrogen. However, the amino acid pools in the mutant responded less dynamic to the altered nutritional status than in the wild type. The observed phenomenon of increasing intracellular amino acids within the first 6 h of nitrogen starvation is explained by the fact that the light harvesting phycobiliproteins are degraded within the first hours of nitrogen starvation, due to the action of activated NblA [22,23]. Prolonged nitrogen starvation decreases the intracellular amino acid pools significantly, since no nitrogen source is present in the growth medium and biosynthetic activity of glutamine synthetase cannot be sustained, therefore biosynthesis of amino acids is halted. The steady decrease of glutamate and glutamine throughout nitrogen starvation is caused by the increased activity of the GS-GOGAT cycle and lack of a nitrogen source. In this process the supply of the cycle with ammonia is interrupted however glutamine and glutamate are still consumed for protein biosynthesis, causing the steady intracellular decline of both amino acids.

Thus, amino acid levels in wild type and *sll0783* mutant behave similarly. These observations confirm our previous results that the mutant is not impaired in acclimation towards nitrogen starvation [11]. Since carbon and nitrogen metabolism are interconnected through the GS-GOGAT cycle we investigated the metabolic changes of the TCA cycle.

#### 2.2.2. TCA-Cycle

**Figure 3:**
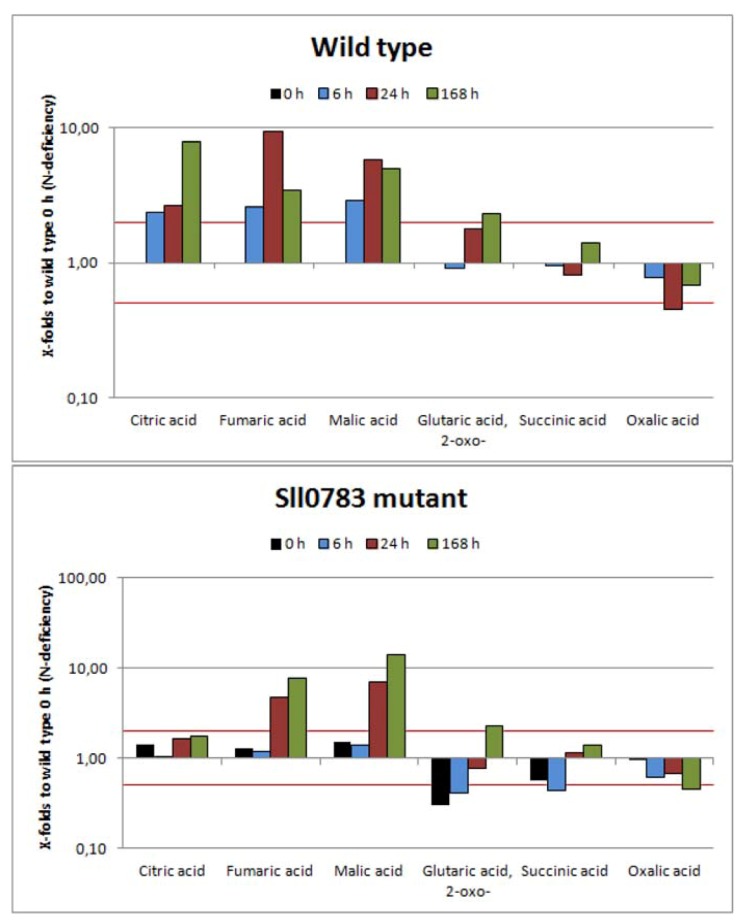
Levels of tri- and dicarboxylic acid intermediates in wild type and the *sll0783* mutant throughout nitrogen starvation. Black bars represent the metabolites levels prior to nitrogen starvation (0 h), cyan bars represent values obtained 6 h, red bars 24 h and green bars 168 h after nitrogen starvation has been induced. All bars represent x-fold changes in metabolite levels compared to the level in wild type at time point 0 h. The red lines mark the values 2 and 0.5. Values above or below these lines represent statistically significant changes of metabolite levels when compared to wild type at time point 0 h.

Figure 3 shows that TCA cycle intermediates accumulated throughout nitrogen starvation in wild type cells. All metabolites with exception of succinic and oxalic acid, which can be derived from the TCA cycle, accumulated in the wild type. Succinic acid levels remained relatively stable and the levels of oxalic acid decreased slightly, which can be derived from oxaloacetic acid. In contrast, however, intracellular concentrations of the other four detected TCA intermediates increased. Levels of citric acid were 9 times higher 168 h after onset of nitrogen starvation compared to levels before nitrogen starvation. This accumulation could be explained by increased NADPH levels, which act as feedback inhibitors of isocitrate dehydrogenase [24], leading to an accumulation of isocitrate. The chemical equilibrium catalyzed by aconitase is strongly on the side of citrate [25], hence citrate accumulates. Fumaric acid levels peaked 24 h after nitrogen starvation was induced. At this time point almost 10 times more fumaric acid was present than prior to nitrogen starvation. However, the intracellular amount of fumaric acid decreased once nitrogen starvation progressed. Similar tendencies were also observed for malic acid. Levels increased throughout nitrogen starvation and peaked 24 h after shift to nitrogen limiting conditions followed by a decrease at 168 h, which was not as profound as seen for fumaric acid. Levels of 2-oxoglutaric acid increased as well but not as dramatically as seen for the other three metabolites described previously.

Compared to wild type, the levels of the TCA cycle intermediates were slightly different in cells of the *sll0783* mutant. Citric acid never accumulated as seen in the wild type and considering the somewhat increased levels prior to nitrogen starvation intracellular levels of citric acid remained almost unchanged throughout the period metabolites were monitored. Initially, levels of 2-oxoglutaric acid and succinic acid were lower than in the wild type but steadily increased throughout nitrogen starvation reaching intracellular concentrations similar to wild type. Intracellular concentrations of oxalic acid behaved similarly to the wild type and only minor differences could be observed. Levels of malic acid and fumaric acid rose in the mutant to levels even higher than seen in the wild type and remained high; however the rise was delayed and observed 24 h and not 6 h after nitrogen depletion. Since precursors for the TCA cycle are generated through glycolysis we also investigated some selected metabolites of this pathway.

#### 2.2.3. Sugar Metabolism

**Figure 4:**
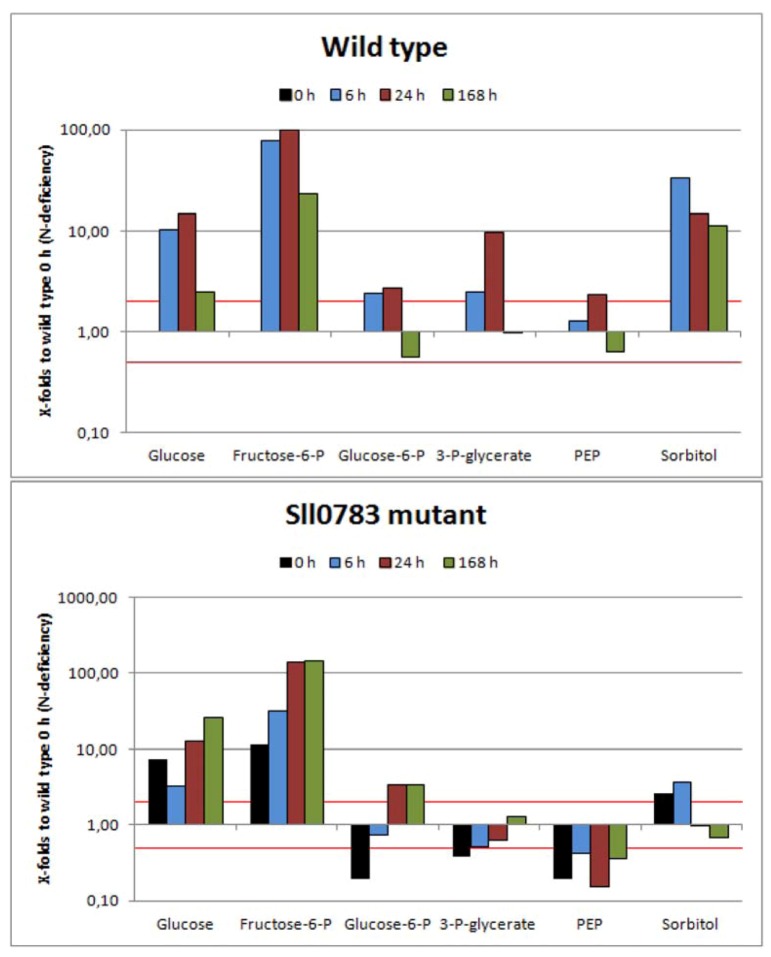
Accumulation of sugar metabolites in wild type and the *sll0783* mutant throughout nitrogen starvation. Black bars represent the metabolites levels prior to nitrogen starvation (0 h), cyan bars represent values obtained 6 h, red bars 24 h and green bars 168 h after nitrogen starvation has been induced. All bars represent x-fold changes in metabolite levels compared to the level in wild type at time point 0 h. The red lines mark the values 2 and 0.5. Values above or below these lines represent statistically significant changes of metabolite levels when compared to wild type at time point 0 h.

Intermediates of sugar metabolism increased within the first 24 h of nitrogen starvation in cells of the wild type, followed by a decrease as nitrogen starvation progressed (Figure 4). Glucose, fructose 6-phosphate and 3-phosphoglycerate levels increased significantly 24 h after depletion of a nitrogen source, however decreased again with prolonged nitrogen starvation. Especially fructose 6-phosphate levels increased up to 100-fold compared to the control. Glucose 6-phosphate levels and phosphoenolperuvic acid levels increased as well, but not as strongly as for the three metabolites mentioned above. The burst of fructose 6-phosphate and 3-phosphoglycerate levels (Figure 4) seems to be an immediate consequence of nitrogen starvation. The latter metabolite is the first stable product of carbon fixation and precursor of most organic carbon in *Synechocystis* [26]. While under nitrogen sufficient conditions, fixed carbon is rapidly converted into PEP to drive amino acid biosynthesis, this can't happen under nitrogen limitation. Instead, 3-phosphoglycerate accumulates and fixed carbon is used for glycogen synthesis through gluconeogenesis [27], explaining the increased glucose and fructose 6-phosphate levels. This interpretation is supported by expression analysis of *Synechocystis* upon nitrogen starvation, which shows that expression of *gap2* (glyceraldehydes-3-phosphate dehydrogenase), an enzyme required for gluconeogenesis [28], and glycogen biosynthetic genes *glgA* and *glgB* are upregulated within 6 h of nitrogen starvation [15], resulting in accumulation of glycogen. A similar tendency in the response of these sugar and glycolysis metabolites was observed for these metabolites in the *sll0783* mutant, however initial metabolite levels differed from wild type. This could be due to the fact that Sll0783 is already expressed prior to nitrogen starvation (see immunoblot analysis of Sll0783 protein in supplementary Figure S4) and a knock out could alter metabolism under nitrogen sufficient growth conditions which cannot be seen phenotypically [11]. A striking difference between wild type and mutant was observed for the change in the intracellular level of sorbitol. In the wild-type, sorbitol levels increased strongly in the first 6 h of nitrogen-starvation (30-fold accumulation) and then remained high throughout nitrogen starvation. In the *sll0783* mutant, sorbitol levels almost did not increase in response to nitrogen-starvation. By contrast, after 24 h sorbitol levels decreased below the initial level. Sorbitol biosynthesis is dependent on a reduction step by which a hexose is reduced by NADPH leading to sorbitol. Obviously, the mutant is not capable of catalyzing this reaction efficiently. This finding was taken as a first hint that the redox balance could be disturbed in the mutant. Probably, mutant cells are less capable of providing sufficient NADPH to drive the sorbitol biosynthesis. A full list of metabolites detected is provided in the supplementary section.

### 2.3. Nitrogen Starvation Shifts the Intracellular Redox Balance towards a More Reduced State

**Figure 5:**
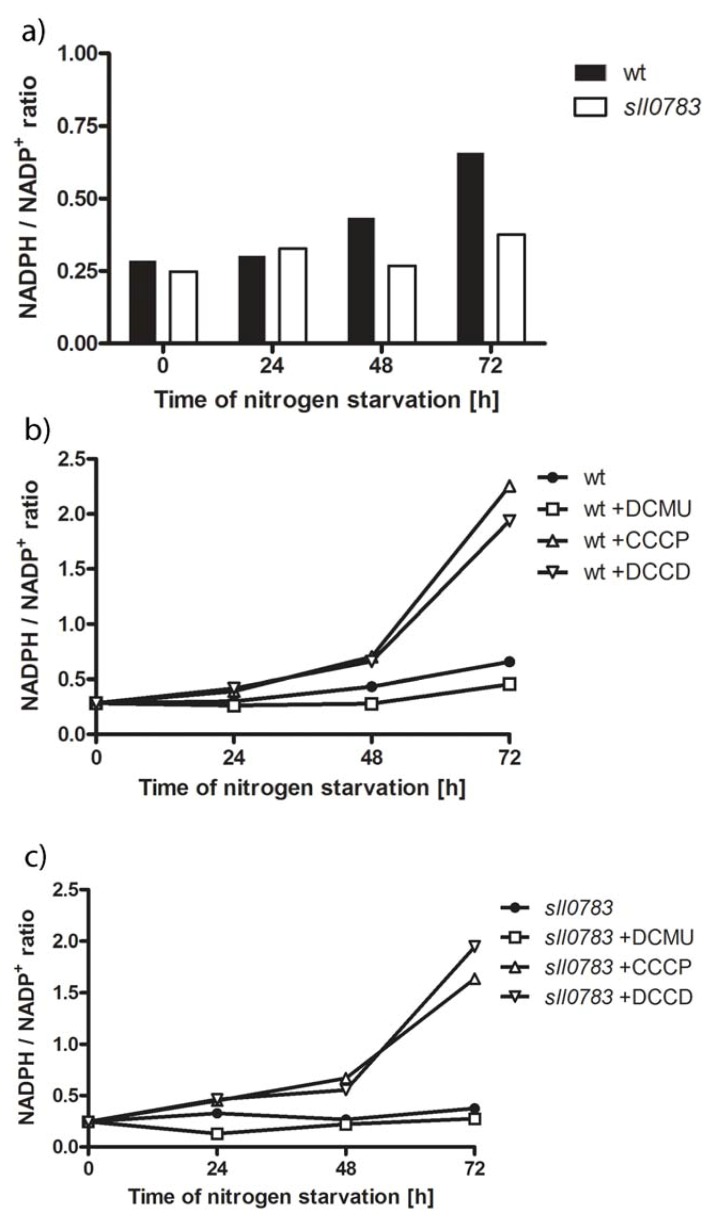
(**a**) The intracellular redox state in wild type (black bars) and the *sll0783* (white bars) changes upon nitrogen starvation. (**b**) Intracellular redox state during nitrogen starvation of the wild type without inhibitor (circle), incubated with DCMU (squares), incubated with CCCP (triangle) and DCCD (inverted triangle). (**c**) Intracellular redox state during nitrogen starvation of the *sll0783* mutant without inhibitor (circle), incubated with DCMU (squares), incubated with CCCP (triangle) and DCCD (inverted triangle). Errors between individual measurements were below 5%; therefore, error bars are left out.

The results from metabolome analysis indicated the observed phenotype of diminished PHB synthase activity and impaired PHB accumulation might be caused by a change in the intracellular redox pool, which has been previously suggested to play a role in regulating PHB biosynthesis [24]. Hence, we determined the ratio between NADPH and NADP throughout nitrogen starvation in wild type and *sll0783* mutant. To do so, cells were transferred to medium lacking a nitrogen source, harvested and lysed in order to determine the NADPH/NADP ratio by a colorimetric test. With prolonged nitrogen starvation, the balance between NADPH and NADP is shifted in the wild type towards NADPH, whereas the ratio remains stable in the *sll0783* mutant (Figure 5a). This result corresponds with the observation that sorbitol doesn't accumulate in the mutant as seen in wild type, which was assumed to be due to a lack of reducing equivalents. This observation raised the question whether the deficient PHB synthase activity of the mutant can be restored by manipulating the intracellular redox balance by applying defined inhibitors such as DCMU, DCCD and CCCP, which alter the turnover of redox equivalents. DCMU inhibits photosystem II by blocking the QB binding site thereby diminishing the linear photosynthetic electron flow, which in turn lowers the intracellular NADPH concentration [29]. CCCP uncouples ADP phosphorylation from the membrane potential thereby generating a lack of ATP for biosynthetic reactions. DCCD specifically inhibits ATP synthase without inhibiting photosynthetic electron flow, which in turn leads to decreased intracellular ATP levels [30]. Low levels of ATP lead to a reduced carbon flow through the Calvin-Benson cycle, which is a major sink for NADPH and ATP in photosynthetic organisms thereby creating a more reducing intracellular environment. All three inhibitors of energy metabolism were added to the cultures after they have been shifted to medium lacking nitrogen and cells were harvested as described in the methods section to determine the NADPH/NADP ratio. DCMU treatment shifted the redox balance slightly towards NADP, whereas DCCD and CCCP strongly shifted the balance towards NADPH, both in wild type and *sll0783* mutant as shown in Figure 5b, c. The shift towards more NADPH than NADP became more profound as nitrogen starvation progressed and was highest after 72 h.

Even though all three inhibitors act immediately on their targets, a time period of 48 h was required to see a profound difference in NADPH between treated and non-treated cells. The delayed response could be explained by the special physiological state the cell enter upon nitrogen starvation. The activity of PSII is down-regulated [3], the phycobiliproteins are degraded and in general, cells tune down metabolic activity [31]. The free amino acids obtained by phycobiliprotein degradation can be catabolized to sustain the redox balance or supply cells with ATP by substrate chain phosphorylation. On the other hand, the cells accumulate the reduced polymers glycogen and PHB, which now act as a sink for reduction equivalents. Altogether, the intracellular redox state appears to be highly regulated and severe metabolic perturbation is required to bring it out of balance [27].

In summary, these measurements confirmed that the used substances were suited to alter the intracellular redox state and gave us the opportunity to test our hypothesis that the observed phenotype of diminished PHB synthase activity and impaired PHB accumulation is caused by the disturbed redox balance in the *sll0783* mutant.

### 2.4. PHB Synthase in the sll0783 Mutant is Impaired Due to an Altered Redox Balance

**Figure 6:**
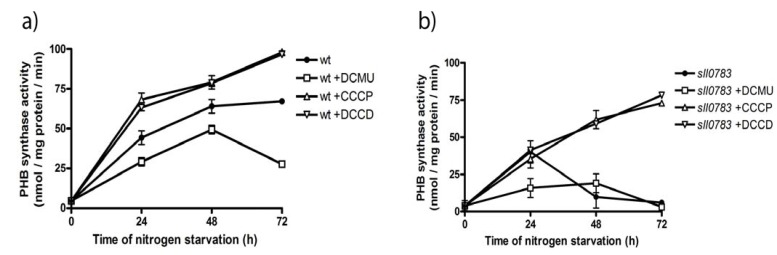
(**a**) PHB synthase activity in crude cell-extracts in wild type cells without inhibitors (circle), incubated with DCMU (squares), incubated with CCCP (triangle) and DCCD (inverted triangle) throughout nitrogen starvation. (**b**) PHB synthase activity in crude cell extracts of *sll0783* mutant cells without inhibitors (circle), incubated with DCMU (squares), incubated with CCCP (triangle) and DCCD (inverted triangle) throughout nitrogen starvation. Values are the mean of three independent measurements.

Addition of DCMU, CCCP or DCCD was previously reported to influence PHB accumulation in the cyanobacterium *Nostoc muscorum* [32] and treatment of *Spirulina maxima* with CCCP resulted in PHB accumulation during nitrogen sufficient conditions [33]. Our measurements showed that these inhibitors affect the intracellular NADPH/NADP ratio (Figure 5). This led to the hypothesis that PHB biosynthetic activity can be recovered in the *sll0783* mutant by altering the intracellular redox balance using these inhibitors. This possibility was tested by shifting the cells into nitrogen-free medium and adding the inhibitors to the cell culture. PHB synthase activity was determined as described in the methods section 24 h, 48 h and 72 h after cells had been shifted to nitrogen free medium. The influence of DCMU, CCCP and DCCD on biosynthetic activity of PHB synthase was significant. Inhibition of the linear photosynthetic electron flow by DCMU markedly decreased PHB synthase activity in wild type and *sll0783* mutant, whereas both inhibitors affecting ATP biosynthesis had the opposite effect as seen in Figure 6a, b. CCCP and DCCD strongly increased biosynthetic activity of PHB synthase in the wild type and *sll0783* mutant to levels which were higher than without inhibitors. Both CCCP and DCCD were able to suppress the phenotype of diminished biosynthetic activity of PHB synthase in the *sll0783* mutant during nitrogen starvation. Biosynthetic activity of PHB synthase was strongly increased and the mutant reached activity values similar to the wild type without any inhibitor. The physiological complementation of the *sll0783* mutant using uncouplers of ATP synthesis, which results in a more reduced intracellular environment, confirms the proposal that the disturbed NADPH pool in the mutant is responsible for impaired PHB synthase activity. Once the redox balance is restored, PHB synthase activity rises to values similar to wild type and thereby physiologically complements the mutant phenotype. Our measurements of the NADPH/NADP ratio (Figure 5) and the PHB synthase activity (Figure 6) also explain why [32] were able to increase PHB accumulation by utilizing these metabolic inhibitors. This highlights the importance of the redox balance to sustain the biosynthetic activity of PHB synthase.

## 3. Experimental Section

### 3.1. Culture Conditions

Wild type *Synechocystis* sp. strain PCC 6803, the *sll0783* mutant and the corresponding reporter strains (harboring pVZ322-1829 or pVZ322-1830) were grown photoautotrophically in BG11 medium [34] supplemented with 5 mM NaHCO_3_ in flasks shaken at 150 rpm at a continuous photon flux density of 50 µmol photons m^−2^ s^−1^ at 28 °C. For initiation of nitrogen deprivation, 50 ml of exponentially growing cells was harvested by centrifugation (8 min, 4,000 rpm) and the pellet was resuspended in NaNO_3_ -free BG11 medium (BG11-N) and centrifuged again. Finally, the washed cells were resuspended again in BG11-N to an optical density at 750 nm (OD_750_) of 0.4 and incubated as described above. To alter the intracellular redox state DCMU, CCCP and DCCD were added to a final concentration of 10 µM in the growth medium. To ensure sufficient carbon supply for PHB biosynthesis, 10 mM acetate was added to the growth medium when PHB biosynthetic assays and the NADPH/NADP ratio were determined.

### 3.2. Metabolite Extraction

Samples of 5 to 10 ml cells, equivalent to approximately 10^9^ cells/ml, were separated from the medium by quick filtration (0.45-mm nitrocellulose filters, Schleicher and Schuell) in the light. Cells on filters were placed in 2-ml Eppendorf tubes and immediately frozen in liquid nitrogen. The metabolomics profiling via GC-EI-TOF-MS, compound identification and data processing were doneaccording to [19].

### 3.3. Measurements of the NADPH Pool

Colorimetric assays were performed using commercial kit NADP^+^/NADPH Quantification Kit (Biovision, USA). Biochemical reactions were stopped by adding equal volumes of ice to the cell culture and cells were pelleted. The cell pellet was suspended in 200 µL NADP^+^/NADPH extraction buffer and disrupted in a Fast-Prep24 apparatus (MP Biomedicals, USA) for 3 × 20 sec with an intensity setting of 6.5 M/s. Cell debris was removed by centrifugation at 1,000 g for 1 min. The supernatant was centrifuged again at 25,000 g for 30 min. Afterwards the supernatants were normalised to protein level before they were passed through a Microcon YM-10 filter (Millipore, USA) to remove NADPH consuming enzymes. The flow-through was used for the detection of both, NADPH and total NADP^+^/NADPH level according to the manual. All samples were processed in a single multiwell experiment run to aid quantification and comparability. Colorimetric measurements were made at 25 °C using optical density measurements at 450nm (OD_450_) in a microplate reader EL808 (BioTek, USA). OD_450_ measurements were converted to ng/mg protein using a standard curve for NADPH.

### 3.4. PHB Synthase Assay

All steps for preparing cell extracts were performed at 4°C or on ice. Cells of *Synechocystis* sp. PCC 6803 and corresponding mutants were harvested from nitrogen-*limited* or nitrogen-sufficient cultures by centrifugation for 8 min at 4,000 g. Then, they were suspended in lysis buffer (25 mM Tris/HCl, pH 7.4, 50 mM KCl, 5 mM MgCl_2_ and 0.5 mM EDTA) and disrupted in a Fast-Prep24 apparatus (MP Biotechnology, Germany) for 3 × 20 sec with an intensity setting of 6.5 M/s. Cell debris was removed by centrifugation at 1,000 g for 1 min to obtain the crude extract. For further analysis, crude extract was separated into a soluble and insoluble fraction by centrifugation at 20,000 g for 15 min. The insoluble fraction was suspended in 0.5 ml Tris/HCl, pH 7.4.

Assay of PHB synthase activity was carried out as described previously [35]. The assay mixtures (200 µL) contained 20 µg of protein, 100 µM DL-3-hydroxybutyryl-CoA, and 1 mM 5,5'-dithiobis (2-nitrobenzoic acid) (DTNB) in 25 mM Tris/HCl, pH 7.4, buffer with 20 mM MgCl_2_. The reaction mixtures were transferred to microplate wells, and the reaction was started by addition of the substrate DL-3-hydroxybutyryl-CoA. The reaction was recorded in an EL808 microplate reader (BioTek) at a temperature setting of 30 °C and the time course of the change in A409 (due to the reaction of the released CoA with DTNB) was monitored for 5 min.

### 3.5. Microscopy

PHB granules in *Synechocystis* sp. PCC 6803 cells were visualized by staining with the fluorescent dye Nile red. To 20 µL of cell culture, 6.6 µL Nile red solution (1 µg mL^−1^ in ethanol) was added. Of this mixture, 10 µL were dropped on glass slides, which had been covered with 1 mL 2% agarose in H_2_O and dried. The cells were analyzed by fluorescence microscopy using a Leica DM5500B microscope with a 100x/1.3 oil objective lens (Leica Microsystems, Germany) and a filter cube with 545/50 nm excitation and 610/75 nm suppression. Pictures were taken with a Leica DFC360FX camera. Fluorescence of eGfp fusion proteins was monitored using a filter cube with 470/40 nm excitation and 525/50 nm suppression.Z-Stacks of 0.1-0.2 µm were recorded and processed with the deconvolution software Leica LAS AF. Standard deconvolution procedure was done by using blind method and 10 iterations.

## 4. Conclusions

Our study is the first to investigate global metabolic changes upon nitrogen starvation covering most metabolites of glycolysis, calvin cycle, TCA cycle and amino acid pools in *Synechocystis* sp. PCC 6803. Our data demonstrate that profound changes happen at the metabolic level once nitrogen starvation is applied (Figure 2, Figure 3, Figure 4). The spiking levels of amino acids 6 h after nitrogen starvation correlates with the degradation of the phycobilisomes [3], a reaction that delivers free amino acids (Figure 2). The following dramatic decrease of intracellular amino acids is in agreement to previous studies, reporting that intracellular C:N ratio changes upon nitrogen starvation from 5:1 to 10:1 [15].

Whereas some TCA cycle intermediates accumulated within the first 24 h followed by a decrease throughout nitrogen starvation (citrate, fumarate and malic acid) or others remained almost unchanged (succinate), citric acid was an exception. Its levels remained high in the wild-type even 168 h after nitrogen starvation was induced. This accumulation can be explained by increased NADPH levels in the wild-type (see above), whereas in the *sll0783* mutant, NADPH levels and hence citrate does not accumulate to that extent. In the absence of combined nitrogen, the oxo acids cannot be converted to amino acids. Instead, the flux of newly fixed carbon is immediately redirected towards gluconeogenesis and the synthesis of reduced carbon products. This metabolic shift is clearly visible regarding the sorbitol levels, which strongly increase in the wild-type. Biosynthesis of sorbitol requires NADPH and the inability of the mutant to accumulate sorbitol agrees with its impaired redox-response. Sorbitol is known from plant metabolism and has only been considered as osmoprotectant in *Synechocystis* sp. PCC 6803 [36], but has so far not been characterized as metabolite in cyanobacteria. The present investigation suggests that rapid sorbitol accumulation after nitrogen-deprivation might function as redox-buffering reaction.

Whereas the synthesis of glycogen and sorbitol appears to be the first response of carbon metabolism upon nitrogen starvation, the activity of PHB synthase increases steadily and reaches its maximum only after 2 days of nitrogen starvation in the wild-type. During this time, the intracellular milieu of *Synechocystis* wild type becomes increasingly reduced, whereas this is not the case in the *sll0783* mutant. This correlates with the activity of PHB synthase during ongoing nitrogen starvation. The initial activation of PHB synthase immediately after nitrogen-starvation is most likely mediated by acetyl phosphate [12] whereas the reducing environment is required to sustain this metabolic reaction. Altering the intracellular redox balance towards a more reducing environment with CCCP and DCCD (Figure 5b, c) increased biosynthetic activity of PHB synthase in wild type beyond the untreated control and restored activity in the mutant (Figure 6). These data imply that PHB synthesis is redox-controlled and the present investigation corroborates previous suggestions [24], that PHB serves as a redox-sink to store excess NADPH during imbalanced metabolic situations.

Altogether we show that the observed phenotype of impaired PHB accumulation in the *sll0783* mutant is caused by the lack of reducing equivalents and is independent of intracellular localization of both PHB synthase subunits. As Sll0783 is homologous to DsrE family proteins which are involved in regulation of intracellular sulfur redox reactions our results support the role of Sll0783 in the regulation of the intracellular redox balance. Unfortunately we are still not able to pin point the function of this protein; however, upregulation upon nitrogen starvation [15] and high abundance of this protein in nitrogen starved cells [6] (see also supplementrary Figure 4) emphasizes its importance. Based on the operon structure and the transcriptional regulation of *sll0783* and its neighboring genes, which encode a nitrile hydrolase (*sll0784*), a protein with homologies to radical SAM superfamily (*sll0785*) and an acyltransferase (*sll0786*), we presume that these proteins might be involved in degradation and utilization of “unusual” nitriles occurring in nature [37]. The specific deletion of *sll0783* might deregulate a redox-chain reaction catalyzed by these proteins, leading to an uncontrolled consumption of NADPH altering the intracellular redox balance and causing the observed phenotype of decreased PHB accumulation.

## Supplementary Files

Supplementary File 1 Supplementary (ZIP, 2222 KB)
