# Supplementary figures and images for: Metabolic Changes in Synechocystis PCC6803 upon Nitrogen-Starvation: Excess NADPH Sustains Polyhydroxybutyrate Accumulation
Source: Metabolites. 2013 Feb 6;3(1):101–18. doi: 10.3390/metabo3010101 (PMC3901256; doi:10.3390/metabo3010101)

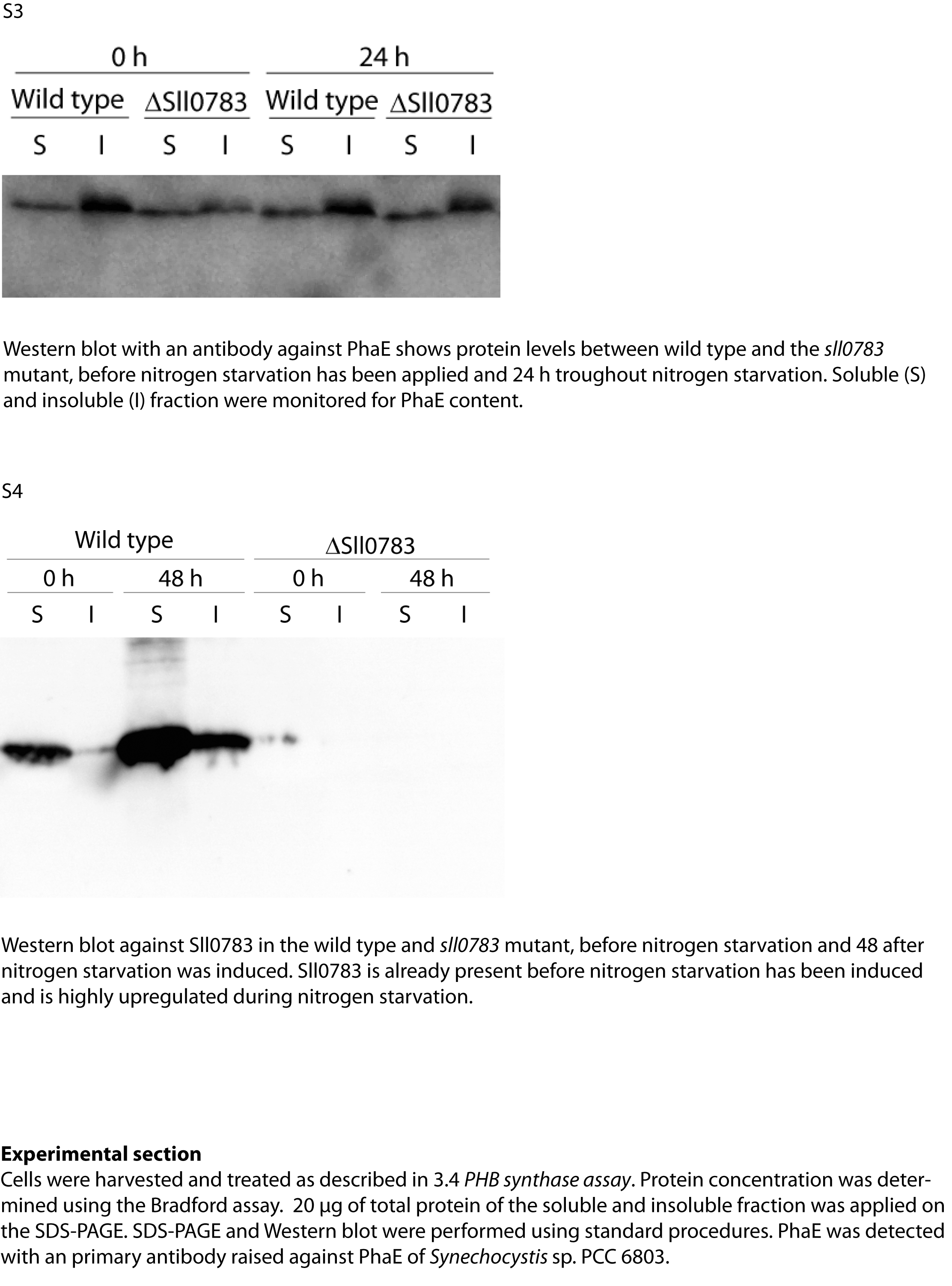

Supplement: Supplementary File 1 — Supplementary (ZIP, 2222 KB) [file metabolites-03-00101-s001.zip › Supplementary Figure 3-4.tif]

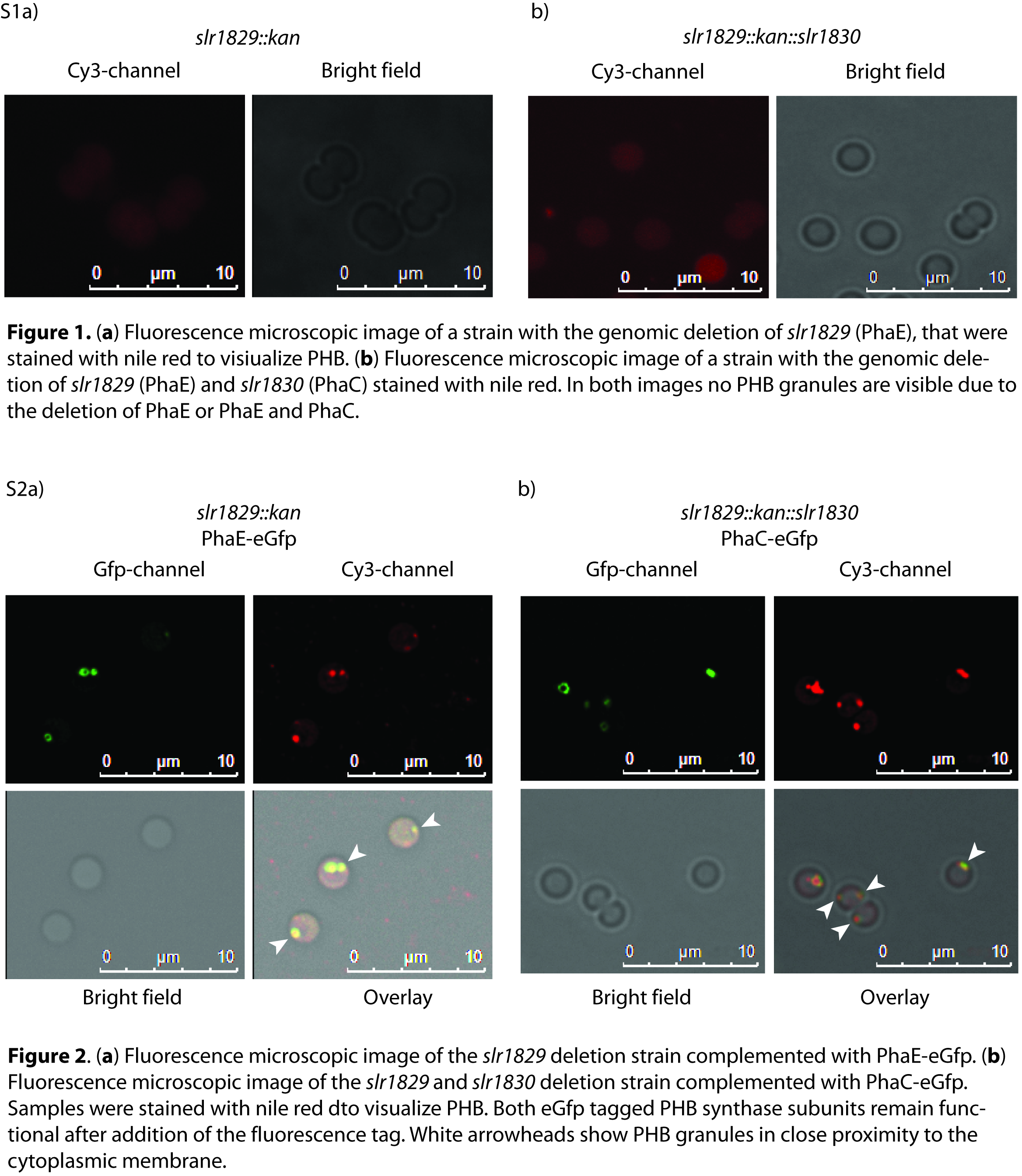

Supplement: Supplementary File 1 — Supplementary (ZIP, 2222 KB) [file metabolites-03-00101-s001.zip › Supplementary figures S1S2.tif]
